# Role of ^18^FDG PET/CT metabolic parameters in predicting hematological toxicity during chemoradiotherapy for locally advanced cervical cancer

**DOI:** 10.3389/fonc.2022.956652

**Published:** 2022-08-18

**Authors:** Tianyu Meng, Xiangxi Meng, Xiaoxia Xu, Xiaofan Li, Zhi Yang, Nan Li

**Affiliations:** ^1^ Key Laboratory of Carcinogenesis and Translational Research (Ministry of Education/Beijing), NMPA Key Laboratory for Research and Evaluation of Radiopharmaceuticals (National Medical Products Administration), Department of Nuclear Medicine, Peking University Cancer Hospital and Institute, Beijing, China; ^2^ Key Laboratory of Carcinogenesis and Translational Research (Ministry of Education/Beijing), Department of Radiation Oncology, Peking University Cancer Hospital and Institute, Beijing, China

**Keywords:** positron emission tomography computed tomography, bone marrow, cervical cancer, toxicity, hematology

## Abstract

**Purpose:**

The aim of this study is to evaluate the value of ^18^FDG PET/CT metabolic parameters in predicting hematological toxicity (HT) during chemoradiotherapy (CRT) for locally advanced cervical cancer (LACC).

**Methods and materials:**

Forty-one patients with LACC undergoing concurrent CRT were retrospectively analyzed. The correlations among age, body mass index, FIGO stage, differentiation, maximum diameter of primary lesion, parametrial invasion, lymph node metastasis, pelvic active bone marrow volume (BM_ACT_), BM_ACT_ volume percentage (BM_ACT_%), maximum standardized uptake value (SUVmax), metabolic tumor volume (MTV), total lesion glycolysis (TLG), and HT were analyzed using hypothesis testing and logistic regression. A p-value< 0.05 was considered significant unless otherwise specified.

**Results:**

Among the 41 patients, 19 had grade 3–4 HT and 22 had grade 0–2 HT. Only SUVmax (Z = −1.961, p = 0.050) and BM_ACT_% (χ2 = 7.769, p = 0.020) showed statistically significant difference in univariate analysis. In logistic regression, grade 3–4 HT was not associated with SUVmax. The probability of HT occurrence in<30% BM_ACT_% was 0.071 times less than in 30%–40% BM_ACT_% (p = 0.010, OR = 0.071, 95% CI = 0.010–0.532), and the probability of HT occurrence in >40% BM_ACT_% was 0.148 times less than in 30%–40% BM_ACT_% (p = 0.037, OR = 0.148, 95% CI = 0.025–0.892).

**Conclusion:**

Baseline ^18^FDG PET/CT BM_ACT_% could help predict the severity of HT during CRT for LACC.

## Introduction

Cervical cancer is the fourth most common cancer among women in the world (1). The standard treatment for locally advanced cervical cancer (LACC) (FIGO stage IB2-IVA) is concurrent chemoradiotherapy (CRT), which can improve the overall survival and local control rate and make the 3-year disease-free survival rate reaching 40%–75% (2, 3). Bone marrow can be divided into red bone marrow and yellow bone marrow. Red bone marrow is mainly distributed in pelvis, lumbar, and thoracic vertebrae and has highly hematopoietic activity. The irradiation and the cytotoxic drugs induce the red bone marrow to differentiate into adipocytes, increase the fat content of bone marrow, and inhibit hematopoietic function (4), resulting in treatment delay, reduction of chemotherapy effectiveness, infection, blood transfusion, etc. (5, 6). Therefore, the prediction of hematological toxicity (HT) of LACC during CRT before treatment is important.

^18^FDG PET/CT has been widely used in active bone marrow imaging (6), but there is no literature reporting the role of PET metabolic parameters such as maximum standardized uptake value (SUVmax), metabolic tumor volume (MTV), and total lesion glycolysis (TLG) in predicting HT during CRT. Moreover, whether and how can PET/CT defined pelvic active bone marrow volume (BM_ACT_) be applied to predict HT remains questionable (7). Therefore, the purpose of this study is to analyze the predictive effect of baseline 18F-FDG PET/CT BM_ACT_ on HT during CRT for LACC.

## Materials and methods

### Subjects

We retrospectively included patients with cervical cancer in our institution from 2015 to 2021. Inclusion criteria are as follows: a) pathologically proved cervical cancer, 2018 FIGO stage IB2-IVA; b) baseline ^18^F-FDG PET/CT image was performed; c) treated with CRT, and medical records available. Exclusion criteria are as follows: a) previous history of other malignancies; b) HT occurred before treatment; c) accompanied by cardiac, liver, or renal dysfunction; and d) pregnancy.

Finally, 41 cases were included, and their baseline characteristics were summarized in **Table 1**.

**Table 1:** Patient characteristics.

Characteristics	n	Percent (%)
Total	41	100
Age	52.6 ± 10.4	–
BMI	22.6 ± 3.0	–
Pathology		
Squamous cell carcinoma	38	92.7
Adenocarcinoma	3	7.3
FIGO stage		
IB2-II	6	14.6
III	32	78.0
IVA	3	7.3
Differentiation		
Low differentiation	7	17.1
Middle differentiation	24	58.5
High differentiation	10	24.4

BMI, body mass index.

### Examination procedures

All patients were fasting for more than 4 h before examination, and the plasma glucose levels were lower than 10 mmol/L. Then, patients were injected with 3.0–3.7 MBq/kg ^18^F-FDG. After rest for about 1 h, the whole-body PET/CT scan was acquired using a Biograph mCT Flow 64 scanner (Siemens, Erlangen, Germany) (120 kV; 310 mAs; slice, 3 mm; matrix, 200 × 200; filter, 5-mm Gaussian), continuous table moving acquisition with a flow speed of 1.5 mm/s over the torso of each subject (from the base of the skull to the middle of the femur). The reconstruction algorithm is ordered subset expectation maximum, iteration 2, subset 21, with point spread function (PSF) and time of flight (TOF) enabled.

### Data analysis

Two experienced nuclear medicine physicians independently reviewed all studies, and disagreements were resolved by consensus. Maximum diameter of primary lesion, parametrial invasion, and lymph node metastasis were analyzed and recorded. The Siemens workstation (Simens Syngo.*via* VB20A) was used for postprocessing. A three-dimensional ellipsoid volume of interest (VOI) was manually drawn to each primary lesion. SUVmax was defined as the maximum SUV within the VOI. The segmentations with SUV greater than 40% SUVmax were used to calculate MTV and TLG, as described in a previous literature (8).

If the interference of other high uptake foci (such as urine) cannot be avoided when drawing VOI of primary lesion, then we used ITK-SNAP software (version 3.8.0) to draw another VOI in the interfered area, used MATLAB software (MathWorks, version r2020a) to clear the PET data in the drawn VOI to remove the interfering volume, and then used the post-processing workstation to obtain PET metabolic parameters, as described above.

**Figure 1:**
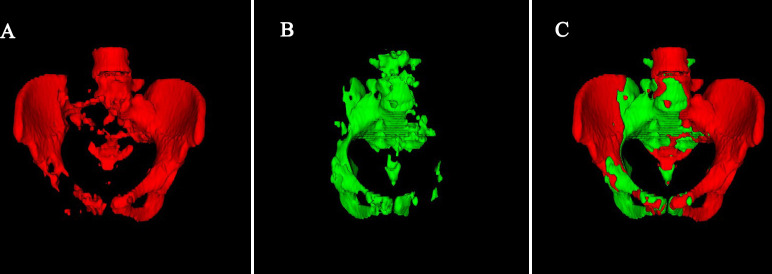
A case of BM_ACT_ and BM_TOT_ distribution. **(A)** Red indicates that bone SUV is lower than the average SUV of the patient’s individual mean SUV. **(B)** Green indicates that bone SUV is greater than or equal to the average SUV of the patient’s individual mean SUV. **(C)** Total volume of Pelvic bone.

Pelvic bone, including L4-L5 vertebrae and bones within the pelvis extending inferiorly to the ischial tuberosity, was drawn by semi-automatic method (9). First, an automatic thresholding segmentation was done by MATLAB to isolate the pelvic bone. In this procedure, Hounsfield unit greater than or equal to 150 was considered as bone. Second, the selected regions were manually corrected. BM_TOT_ volume was define as the volume of the pelvic bone. BM_ACT_ for each patient was defined as volume of the region within the BM_TOT_ with an SUV greater than or equal to the patient’s individual mean SUV. BM_ACT_% was defined as BM_ACT_ divided by BM_TOT_. A case of BM_ACT_ and BM_TOT_ distribution is shown in **Figure 1**.

### Treatments

All patients received concurrent CRT. IMRT or rapid arc intensity-modulated radiotherapy (RapidArc) technology was used for external irradiation. The total pelvic radiotherapy was 45 Gy/25 times. The dosage of pelvic wall was determined according to the FIGO stage. 192Ir brachytherapy treatment was performed four to six times after 3 weeks of external irradiation, and the total dose was 75~85 Gy. All chemotherapy regimens were platinum-based. The regimens included TP (D1: paclitaxel, 135 mg/m^2^; D2: cisplatin, 60 mg/m^2^; 21-day repeat, one to two cycles), weekly cisplatin (D1: 40 mg/m^2^, four to six cycles).

### Hematologic toxicity

A complete blood cell count was given to the patient before any treatment and then weekly during the CRT. The frequency of complete blood cell count can be increased if necessary. In this study, two patients received blood transfusion due to low platelet count: one was given an additional complete blood cell count after blood transfusion, and the other was given two additional complete blood cell counts after blood transfusion. Nadirs of absolute counts of white blood cells (WBC), neutrophils (ANC), hemoglobin (HGB), and platelets (PLT) were recorded and graded according to Common Terminology Criteria for Adverse Events (CTCAE) version 5 to determine leukopenia, neutropenia, anemia, and thrombocytopenia. Hematologic toxicity was defined as the maximum grade of these hematological abnormalities. Patients were grouped according to more severe than grade 3 HT (recorded as G3+ group) and grade 0–2 HT (recorded as G0–2 group).

### Statistical analysis

All statistical tests were conducted using IBM SPSS version 22.0 for Windows (IBM Corp, Armonk, NY, USA). The difference between baseline and nadir was compared by paired t-test (accords with normal distribution) or Wilcoxon signed-rank test (not accords with normal distribution). The normality testing was conducted using Kolmogorov–Smirnov testing. The Chi-square test or Fisher exact test was used to compare the categorical variables. The Student t-test was used for those accord with normal distribution, and the Mann–Whitney U-test was used for those not accord with normal distribution. Then, the binary logistic regression was used to determine the relationship between HT and factors with statistically significant differences in the univariate analysis (corresponding to a p-value less than 0.1). Holm–Bonferroni correction was used in multi-group comparison. A p-value less than 0.05 was considered statistically significant in paired t-test, signed-rank test, and hypothesis testing in the logistic regression.

## Result

### Changes in peripheral blood count during treatment

**Figure 2:**
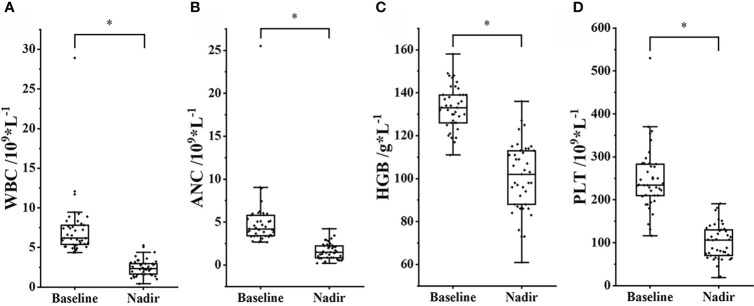
Changes in peripheral blood cell count between baseline and nadir. **(A)** WBC. **(B)** ANC. **(C)** HGB. **(D)** PLT. *, *p<* 0.05.

The mean values of WBC, ANC, PLT, and HGB declined significantly during treatment. The nadir of WBC was 2.47 ± 1.05, and the baseline was 7.27 ± 3.91, *p<* 0.001; the nadir of ANC was 1.61 ± 0.95, and the baseline was 5.12 ± 3.60, *p<* 0.001; the nadir of HGB was 101 ± 16, and the baseline was 133 ± 10, *p<* 0.001; the nadir of PLT was 101 ± 41, and the baseline was 248 ± 73, *p<* 0.001, as shown in **Figure 2**.

During CRT, a total of 19 patients (46.3%) had grade 3–4 HT, the remaining 22 patients (53.7%) had grade 0–2 HT, and no patient had grade 5 HT. Among them, 14 cases (34.1%) had grade 3–4 leukopenia, 13 cases (31.7%) had grade 3–4 neutropenia, four cases (9.8%) had grade 3–4 anemia, and three cases (7.3%) had grade 3–4 thrombocytopenia, as shown in **Table 2**.

**Table 2:** Hematologic toxicity during chemoradiotherapy.

Toxicity	Grade (%)				
	0	1	2	3	4
All	2 (4.9)	2 (4.9)	18 (43.9)	15 (36.6)	4 (9.8)
Leukopenia	3 (7.3)	7 (17.1)	17 (41.5)	13 (31.7)	1 (2.4)
Neutropenia	14 (34.1)	6 (14.6)	8 (19.5)	10 (24.4)	3 (7.3)
Anemia	14 (34.1)	7 (17.1)	16 (39.0)	4 (9.8)	0 (0.0)
Thrombocytopenia	21 (51.2)	7 (17.1)	10 (24.4)	1 (2.4)	2 (4.9)

### Relationship of various factors with HT

The differences in clinical and pathological features [including age, body mass index (BMI), FIGO stage, differentiation, maximum diameter of primary lesion, parametrial invasion, and lymph node metastasis] and PET/CT metabolic parameters (including BM_ACT_, SUVmax, MTV, and TLG) between the two groups were compared. There was a significant difference in SUVmax between G3+ group and G0–2 group (*Z* = −1.961, *p =* 0.050), whereas there was no significant difference in other variables between the two groups (*p* > 0.1). When BM_ACT_% was included in the analysis as a continuous variable, there was no significant difference between G3+ group and G0–2 group (*t* = −0.496, *p =* 0.623). However, when BM_ACT_% was grouped as< 30%, 30%–40% and > 40%, there was a significant difference between G3+ group and G0–2 group (*χ2 =* 7.769, *p =* 0.020); see **Table 3** for details. The Chi-square test found that, in G3+ group and G0–2 group, BM_ACT_%< 30% *vs*. 30%–40% (*χ2 =* 7.538, *p =* 0.006) and BM_ACT_% 30%–40% *vs*. > 40% (*χ2 =* 3.877, *p =* 0.049) were significantly different, whereas BM_ACT_%< 30% *vs*. > 40% was not significantly different (*χ2 =* 0.303, *p =* 0.582).

**Table 3:** Univariate analysis of HT during chemoradiotherapy.

HT				
Factors	G0–2 group	G3+ group	χ^2^/t/Z	p
n	22	19	N/A	N/A
Age^a^	52.91 ± 11.47	52.16 ± 9.37	0.227	0.821
BMI^a^	22.45 ± 3.14	22.73 ± 2.95	−0.290	0.774
FIGO stage			0.991	0.635
IB2-II	4	2		
III	17	15		
IVA	1	2		
Differentiation			3.634	0.117
Low differentiation	6	1		
Middle differentiation	12	12		
High differentiation	4	6		
Maximum diameter	4.83 ± 1.45	5.29 ± 1.89	−0.884	0.382
Parametrial invasion			1.154	0.283
Yes	14	15		
No	8	4		
Lymph node metastasis			0.149	0.699
Yes	15	14		
No	7	5		
$BM_{ACT}^{a}$	138.54 ± 53.18	150.83 ± 55.39	−0.724	0.473
*BM*_*ACT*_ *%* ^*a*^	34.64% ± 9.63%	36.11% ± 9.34%	−0.496	0.623
grouped BM_ACT_%			7.769	0.020*
<30%	10	3		
30%–40%	3	10		
>40%	9	6		
Other metabolic parameters				
SUVmax^b^	13.87 ± 7.01	18.93 ± 9.68	−1.961	0.050*
MTV^a^	34.64 ± 26.62	36.40 ± 29.85	−0.201	0.842
TLG^a^	317.88 ± 274.84	422.95 ± 384.62	−1.016	0.316

*, Statistically significant; a, use Student t-test; b, use Mann–Whitney U-test.

### Logistic regression

**Figure 3:**
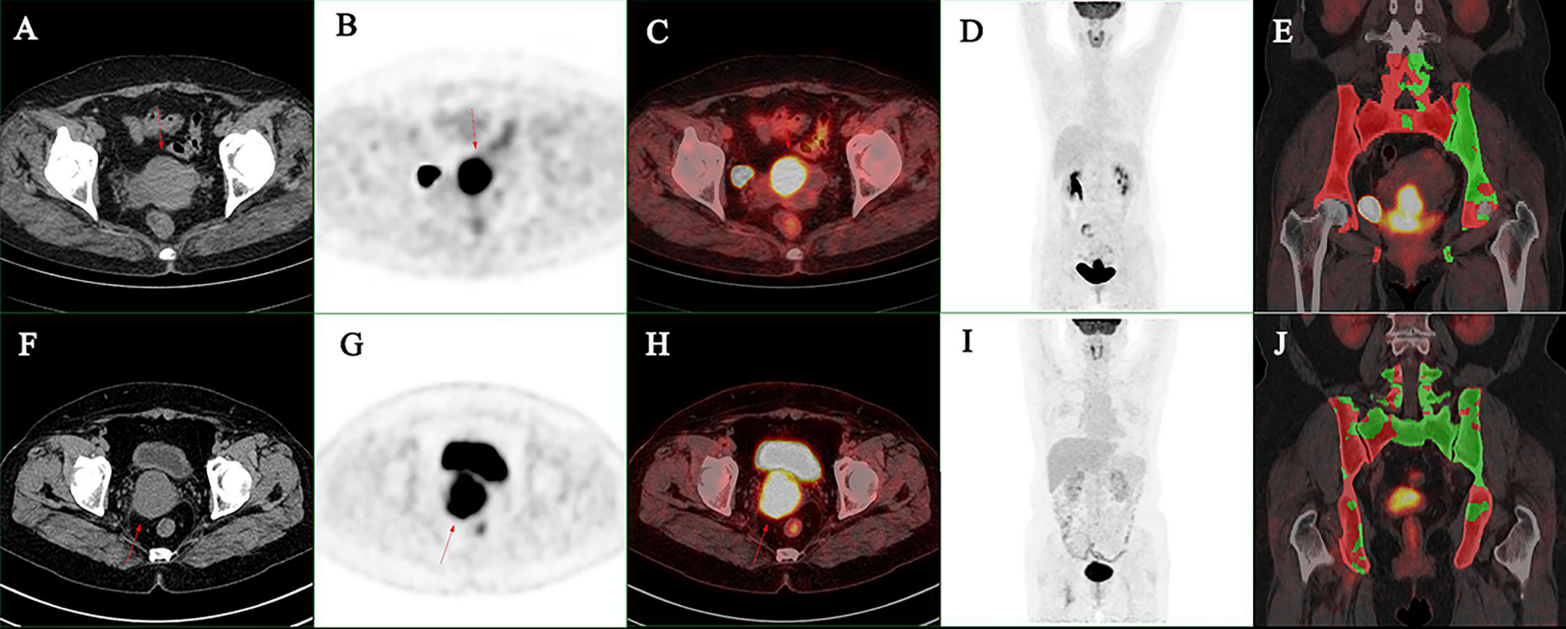
**(A–E)** A 47-year-old women suffered FIGO stage IIIC1r cervical cancer, the maximum diameter of primary lesion was 6.0 cm: the SUVmax was 17.5, the MTV was 39.4 cm^3^, and the BM_ACT_% was 31.2%. G3+ hematologic toxicity occurred during chemoradiotherapy. **(F–J)** A 55-year-old women suffered FIGO stage IIIC1r cervical cancer: the maximum diameter of primary lesion was 5.1 cm, the SUVmax was 12.9, the MTV was 39.5 cm^3^, and the BM_ACT_% was 49.8%. G3+ hematologic toxicity occurred during chemoradiotherapy. **(A, F)** Axial CT image of primary lesion. **(B, G)** Axial PET image of primary lesion. **(C, H)** Axial PET/CT fusion image of primary lesion. **(D, I)** Maximum intensity projection of trunk PET. **(E, J)** Coronal PET/CT fusion image with BM_ACT_ shows in green.

SUVmax and grouped BM_ACT_% were included to develop a logistic regression model. It was found that SUVmax had no predictive effect on HT during radiotherapy and chemotherapy (*p =* 0.059, *OR =* 1.107, *95% CI* = 0.996–1.230). Patient whose BM_ACT_% less than 30% has a probability of occur grade 3+ HT 0.071 times less than that patient whose BM_ACT_% between 30% and 40% (*p =* 0.010, *OR =* 0.071, *95% CI* = 0.010–0.532), and patient whose BM_ACT_% greater than 40% has a probability of occur grade 3+ HT 0.148 times less than that patient whose BM_ACT_% between 30% and 40% (*p =* 0.037, *OR =* 0.148, *95% CI =* 0.025–0.892), respectively (see **Table 4** for details) (see **Figure 3** for typical cases).

**Table 4:** Logistic regression results.

	*p*	OR	95%CI
SUVmax	0.059	1.107	0.996–1.230
30%< BM_ACT_%< 40%	0.028*		
BM_ACT_%< 30%	0.010*	0.071	0.010–0.532
BM_ACT_% > 40%	0.037*	0.148	0.025–0.892

*, Statistically significant.

## Discussion

Concurrent CRT is the main treatment for LACC, but it usually induces some adverse effects. According to the CTCAE standard, toxicity above grade 3 requires clinical intervention, which usually leads to delayed chemotherapy or interruption of radiotherapy (10). Therefore, early prediction of severe HT has important clinical significance. In this study, HT of grade 3 or higher was considered as the criterion of severe toxicity. Previous literature reported that, during CRT for LACC, the proportion of severe HT was about 10%–70% (10). In this study, the proportion is 37%, which is consistent with previous literature reports.

Traditional clinical characteristics have limitations in predicting HT during CRT for LACC. Bosque et al. (11) retrospectively analyzed 59 cases of LACC and found that the group with grade 2 or higher HT during CRT has a significantly lower BMI than that of the group with grade 0–1 HT (*p =* 0.004). Xiang et al. (7) analyzed 184 cases of cervical cancer and revealed that the diagnosed age, FIGO stage, differentiation, and BMI were not related to HT during CRT. In this study, we found that there were no significant differences in BMI between the groups with grade 3–4 HT and grade 0–2 HT. The possible reason is that the BMI of patients included in this study is mostly within the normal range, whereas there are more cases of overweight and obesity in the study of Bosque et al. In this study, age, FIGO stage and differentiation shows no statistically significant in patients with grade 3–4 HT and grade 0–2 HT, which is consistent with Xiang.

CT, MRI, and PET/CT are widely used in staging, radiotherapy planning, response evaluation, and recurrence evaluation of cervical cancer (8, 12, 13), but the ability of these methods to predict HT during CRT for LACC has not been reported in the literature. This study did not find any relation between common PET metabolic parameters, such as maximum diameter of primary lesions, SUVmax, MTV and TLG, and G3+ HT during CRT. The reason may be that, according to the guidelines, the regimen of CRT is unified and is not affected by characteristics of the primary lesion.

The predictive value of BM_ACT_ in predicting HT during CRT has been reported in many literatures. Some studies have analyzed BM_ACT_ radiation dose and HT, but the studies are scarce and the sample sizes are small. For example, Zhou et al. (14) retrospectively studied 31 patients and found that the absolute volume of pelvic active bone marrow spared 40 Gy< 738 cc maybe a stronger predictor of HT, with a sensitivity of 75% and a specificity of 100%. On the other hand, there is no consensus reached in the optimal bone marrow dose/volume limit standard (14, 15), and only few literature studied the relationship between BM_ACT_ and HT. Wang et al. (16) found that, in 39 patients with cervical cancer, the BM_ACT_ determined by ^99m^Tc-sulfur colloid SPECT<387.5 cm^3^ can predict HT, with a sensitivity of 84.2% and a specificity of 85%. Khullar et al. (17) retrospectively studied 21 patients and found that BM_ACT_ determined by ^18^FDG PET/CT< 1201 ml can predict grade 3+HT, with a sensitivity of 66.6% and a specificity of 100%. The sample sizes of these studies were all small, and the conclusions are different. In this study, we found the relationship between the BM_ACT_% defined by baseline PET/CT and HT during CRT seemed to be nonlinear for the first time. When the BM_ACT_% lies between 30% and 40%, the probability of HT was significantly higher than the BM_ACT_% > 40% group, as we expected. However, we found that the incidence of HT in BM_ACT_%< 30% group was lower than 30%–40% group, whereas it was not significantly different from BM_ACT_% > 40% group. The possible reason maybe that, because all the patient had no HT before treatment, the baseline bone marrow reserve was sufficient. When pelvic BM_ACT_% is low, more active bone marrow will be in the rest of the torso, which is not affected by radiotherapy. When BM_ACT_% is high, there will be more residual active red bone cells in pelvic bone during the therapy, and when BM_ACT_% is within a certain range, the hematopoiesis will be greatly affected by CRT. This conclusion needs to be proved by further large sample research studies.

This study has several limitations. First, this was a single-center retrospective study, which may lead to selection bias; second, the sample size of this study is small, and there may be some random errors; and third, the subgroup cutoff of BM_ACT_% also has to be validated by a larger sample size, which requires future studies.

## Conclusion

In conclusion, baseline ^18^FDG PET/CT BM_ACT_% could help predict the severity of HT during CRT for LACC. Other clinical features, pathological features and PET/CT metabolic parameters could not help predict the severity of HT.

## Data availability statement

The raw data supporting the conclusions of this article will be made available by the authors, without undue reservation.

## Ethics statement

Written informed consent was not obtained from the individual(s) for the publication of any potentially identifiable images or data included in this article.

## Author contributions

TM, XM, XL and NL contributed to the study’s conception and design. Material preparation, data collection, and analysis were performed by TM, XX, and NL. The statistical methods were reviewed by XM. The first draft of the manuscript was written by TM and was revised by NL and ZY. All authors contributed to the article and approved the submitted version.

## Funding

This study was funded by Beijing Hospitals Authority’ Ascent Plan, Code: DFL20191102; National Natural Science Foundation (No. 81871387 and No. 81871386); and Beijing Natural Science Foundation (No. 7202027).

## Conflict of interest

The authors declare that the research was conducted in the absence of any commercial or financial relationships that could be construed as a potential conflict of interest.

## Publisher’s note

All claims expressed in this article are solely those of the authors and do not necessarily represent those of their affiliated organizations, or those of the publisher, the editors and the reviewers. Any product that may be evaluated in this article, or claim that may be made by its manufacturer, is not guaranteed or endorsed by the publisher.
